# Insect Community Diversity in Photovoltaic Power Station and Its Response to Environmental Factors

**DOI:** 10.3390/biology14101388

**Published:** 2025-10-11

**Authors:** Ying Wang, Yuanrun Cheng, Liping Ban, Xuewei Yin, Shuhua Wei, Wei Sun, Rong Zhang

**Affiliations:** 1 Institute of Plant Protection, Ningxia Academy of Agriculture and Forestry Sciences, Yinchuan 750002, China; cyr20010216@163.com (Y.C.); weishuhua666@163.com (S.W.); swlymyy@163.com (W.S.); 2 College of Grassland Science and Technology, China Agricultural University, Beijing 100193, China; lipingban@cau.edu.cn (L.B.); stone856@163.com (X.Y.)

**Keywords:** PV station, desert steppe, insect community diversity, biodiversity conservation

## Abstract

To investigate the impact of PV power station construction on insect community diversity in the desert steppe of Ningxia and its response to environmental factors, insect communities were surveyed in different areas within the PV station (both under and between PV panels) and outside the station. The species composition, diversity differences, and responses to environmental factors of insect communities in these areas were analyzed. The results showed that a total of 19,833 insect specimens, belonging to 68 species and 23 families, were collected across different areas of the PV station. The dominant species within the PV station (both under and between PV panels) were *Labidura riparia japonica* (Dermaptera: Labiduridae), *Harpalus sinicus* (Coleoptera: Carabidae) and *Harpalus calceatus* (Coleoptera: Carabidae), while outside the station, the dominant species were *L. r. japonica*, *H*. *sinicus*, *H*. *calceatus* and *Harpalus pallidipennis* (Coleoptera: Carabidae). The number of species by feeding habit ranked as follows: phytophagous insects > predatory insects, whereas the abundance of individuals followed the order: predatory insects > phytophagous insects. The species richness, abundance, Margalef richness index, Shannon–Wiener index and Pielou evenness index of phytophagous insects were significantly higher outside the PV power station than inside (both under and between PV panels). In contrast, Simpson dominance index was significantly lower outside the PV power station compared to inside (both under and between PV panels). For predatory insects, no significant differences were observed in species richness, Margalef richness index, Shannon–Wiener diversity index, Simpson dominance index, or Pielou evenness index among different PV panel areas. However, the abundance of predatory insects was significantly higher outside the PV power station than inside (both under and between PV panels); phytophagous insects in the PV station were primarily positively driven by soil nutrients (total nitrogen, available potassium), whereas predatory insect diversity was more responsive to soil organic matter and nitrogen levels. Both predatory and phytophagous insects showed a significant negative correlation with vegetation height. This study holds significant importance for exploring biodiversity conservation within PV power stations, providing a scientific basis for the planning, design, and implementation of ecological protection measures during the operation of PV station in Ningxia’s desert steppe.

## Simple Summary

The variation in insect diversity serves as a sensitive indicator of environmental disturbance, and studying the impact of photovoltaic (PV) power station construction on insect diversity is crucial for assessing its long-term ecological effects. This study investigated the diversity of insect communities and their driving mechanisms in a desert steppe PV power station in Ningxia. By analyzing the composition and diversity communities across different areas of the PV power station, we further elucidated the key drivers affecting the diversity of insect groups with varying feeding habits. Our results demonstrate that the observed differences are determined by the synergistic effects of multiple environmental factors.

## 1. Introduction

Energy serves as the cornerstone of human societal development, while the utilization of renewable energy represents a critical pathway to achieving the “dual carbon” goals. PV power generation has experienced rapid global expansion in recent years due to its advantages of being clean, efficient, and flexible [1,2]. The arid northwest region of China, endowed with abundant solar resources, has emerged as a prime location for large-scale PV power station development [3]. However, the construction and operation of PV station may alter local ecological conditions, including surface temperature, humidity, soil properties, and vegetation coverage [4], thereby affecting biodiversity—particularly for environmentally sensitive insect communities [5]. Studies have shown that PV power influence soil and vegetation properties, which in turn affect insect physiology and distribution [6]. Previous studies indicate that PV panels provide shade, reduce soil water evaporation, and enhance moisture retention, leading to higher soil water content beneath panels compared to outside the station [7]. Additionally, organic carbon and ammonium nitrogen levels are elevated between PV panels, enhancing soil carbon sequestration while reducing total phosphorus, nitrogen, and potassium content. However, available nutrients increase, promoting plant growth and indirectly benefiting insect development [8]. Consequently, while promoting renewable energy development, investigating the impact mechanisms of PV station on ecosystems holds significant importance for achieving sustainable development.

Located in the arid-semiarid transition area of northwestern China, Ningxia Hui Autonomous Region features temperate desert steppe as its predominant natural grassland type [9,10]. Covering 1.34 million hectares, these grasslands account for 55% of the region’s total grassland area, constituting an ecologically fragile system highly sensitive to environmental changes [11]. In recent years, Ningxia has experienced rapid growth in renewable energy installation capacity. By 2025, PV power generation has come to represent a significant proportion of the Ningxia’s electricity mix [12]. However, the large-scale construction of PV power stations may lead to several ecological impacts including land cover modification, enhanced electromagnetic radiation, and increased microclimate heterogeneity, all of which could potentially affect surface-dwelling arthropods, particularly insects [13]. As crucial components of ecosystems, insects demonstrate high sensitivity to environmental disturbances through their diversity patterns [14]. Therefore, investigating the impacts of PV power station on insect communities provides critical insights for assessing their long-term ecological effects.

Currently, research on the ecological impacts of PV power station, both domestically and internationally, has primarily focused on vegetation restoration [15] and avian conservation [16], while systematic investigations into the mechanisms affecting insect diversity remain scarce. Particularly in desert steppe ecosystems, it remains unclear how PV station operations alter insect communities through changes in electromagnetic fields, noise levels, soil properties, and vegetation structure. Furthermore, the differential responses of various functional groups (e.g., phytophagous and predatory insects) to environmental factors require further in-depth study. Against this backdrop, this study examines phytophagous and predatory insects at the NEG Ningdong 200 MW PV Power station in Lingwu City, Ningxia, to investigate the effects of PV station construction on insect diversity in desert steppe ecosystems. Additionally, the underlying mechanisms—including electromagnetic radiation, noise, soil, and vegetation—are analyzed. The findings aim to provide a theoretical basis for the eco-friendly design of PV station in desert steppe regions and offer scientific support for reconciling renewable energy development with biodiversity conservation.

## 2. Materials and Methods

### 2.1. Monitoring Area

**Figure 1:**
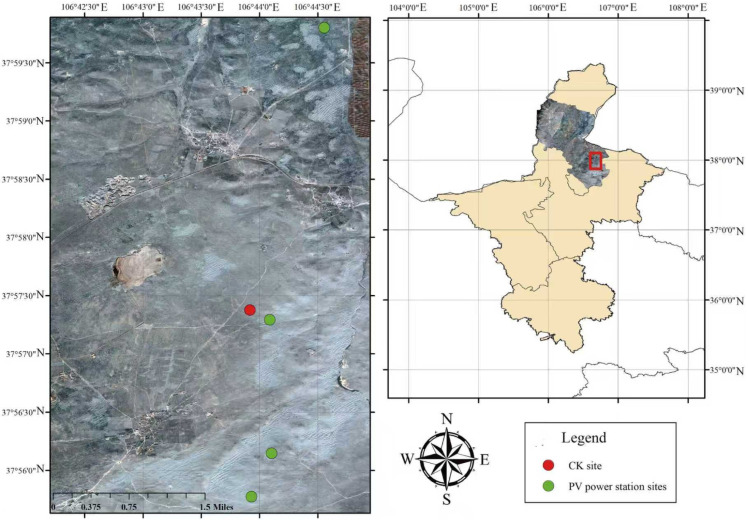
Location map of sampling plots in the PV power station.

**Figure 2:**
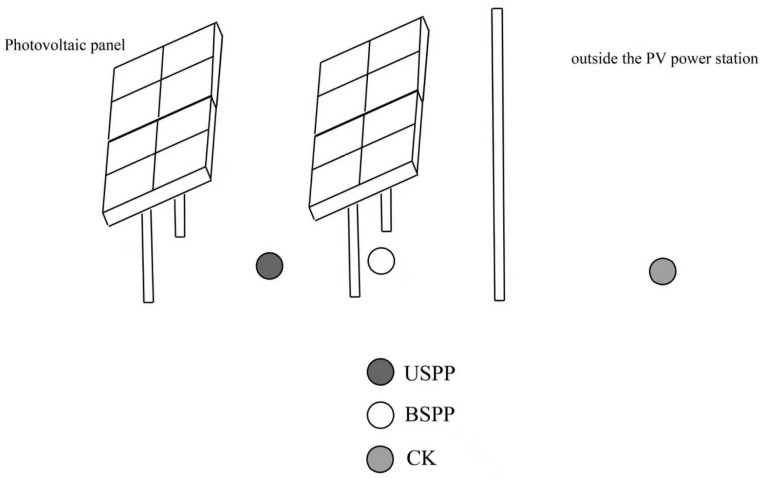
Schematic diagram of sampling points in different areas of the PV power station. Note: USPP is under solar PV panels, BSPP is between solar PV panels, and CK is outside the PV power station.

The study site is located at the Guoneng Ningdong 200 MW photovoltaic base in Lingwu City, Ningxia, covering an area of approximately 6.67 km^2^. The facility includes a 330 kV booster station, nearly 70 km of transmission lines, and an energy storage station with a capacity of 200–400 MWh. The project was substantially completed in early 2023. Ecologically, the area belongs to a temperate desert steppe ecosystem, dominated by *Convolvulus tragacanthoides* (Solanales: Convolvulaceae), *Lespedeza davurica* (Fabales: Fabaceae), and *Artemisia ordosica* (Asterales: Asteraceae). Four survey areas were established within the PV power station, with one control area set up outside the station (Figure 1). Three sampling points were designated at each survey area—both beneath and between the PV panels inside the power station, as well as outside the station (Figure 2).

### 2.2. Survey Methodology

#### 2.2.1. Insect Species Survey Methods

The surveys were conducted in May, July, and September from 2023 to 2024. At each sampling point within the survey areas, three sampling sites were established for fixed-point sweep net surveys and trap collections. Surveys and trapping were conducted once per month.

Sweep Net Method: At each sampling site, 30 sweep repetitions were performed (one repetition consisted of sweeping the insect net close to the vegetation, horizontally 180° to the left and right). The collected insects were placed into a net bag. The insects in the net bag were brought back to the laboratory for counting and made into specimens for identification. The distance between each sampling site was approximately 50 m.

Pitfall Trap Method: Disposable plastic cups (7.5 cm in diameter, 9 cm in height) were used as traps. The cups were entirely buried in the soil, with the rim levelled as much as possible with the undisturbed ground surface. The cups were filled with trapping solution to about one-third of their volume. The trapping solution consisted of 33.33% by mass fraction ethylene glycol and water (in a ratio of ethylene glycol to water = 1:2). At each sampling site, 5 to 10 replicates were set up according to the terrain. The traps were arranged in a straight line, with a distance of more than 2 m between each trap. Samples were collected 10 days after deployment. During collection, the samples were immersed in pure alcohol in collection bottles according to the sampling points for preservation. They were then brought back to the laboratory for counting and made into specimens for identification.

#### 2.2.2. Soil Factor Investigation Methodology

Soil samples from 0–20 cm depth were collected using the five-point sampling method in each survey area. The measured soil physicochemical properties included: Soil pH, Soil organic matter, Total nitrogen, Total phosphorus, Total potassium, Alkali-hydrolyzable nitrogen, Available potassium, Available phosphorus, Electrical conductivity of soil leachate, Soil water content [17].

#### 2.2.3. Electromagnetic Radiation and Noise Measurement Methods

Simultaneously with insect surveys, electromagnetic radiation and noise measurements were conducted at three sampling points: within the PV power station (under and between the panels) and outside the station, with three replicates per sampling point. An electromagnetic radiation detector (Shanghai Yixing Electromechanical Equipment Co., Ltd., Shanghai, China, model TES-593R) and a noise meter (Hangzhou Aihua Instruments Co., Ltd., Hangzhou, China, model TA652A) were used for the measurements.

#### 2.2.4. Vegetation Factor Survey Methods

Within the PV power station and outside the PV power station, two investigation points were set up. Vegetation sampling was conducted using a random sampling method, with a distance of no less than 50 m between quadrats. Three 1 m × 1 m quadrats were randomly established within each monitoring area. Recorded vegetation parameters included: species composition, plant height, coverage (measured using the pin-point method), and frequency (measured using the circle method). All vegetation within each quadrat was cut at ground level and brought back to the laboratory for the determination of aboveground biomass.

### 2.3. Data Statistical Analysis

The composition of insect species was characterized using abundance (N) and species richness (S). Based on the proportion of each species relative to the total abundance, insect species were classified into three categories: dominant species (proportion ≥ 10%), common species (1% ≤ proportion < 10%), and rare species (0 ≤ proportion < 1%). Insect diversity was assessed using the Margalef richness index (d), Simpson dominance index (λ), Shannon–Wiener index (H’), and Pielou evenness index (E), calculated as follows:

Margalef richness index: d = (S − 1)/ln(N)(1)

(2) $$\mathrm{Simpson}\;\mathrm{dominance}\;\mathrm{index}:\;\lambda =\sum \left(\frac{Ni(Ni-1)}{N(N-1)}\right)$$

(3) $$\mathrm{Shannon}\text{–}\mathrm{Wiener}\;\mathrm{index}:\;H^{\prime }=-\sum \left(Pi\times ln\left(Pi\right)\right)$$

(4) $$\mathrm{Pielou}\;\mathrm{evenness}\;\mathrm{index}:\;E=H^{\prime }/ln\left(S\right)$$

S is species richness, N is total abundance, Ni is abundance of the species, Pi is proportion of the species relative to total abundance [18].

Data were processed using Microsoft Excel 2018. Differences in abundance, species richness, and diversity indices were analyzed using one-way ANOVA in SPSS 27.0.1. Data visualization was performed using GraphPad Prism 9.5.0 and Origin Pro 2021. Additionally, we comprehensively employed Non-metric Multidimensional Scaling (NMDS), Principal Component Analysis (PCA), Canonical Correspondence Analysis (CCA), and Generalized Additive Models (GAM) to reveal community differences [19,20], species-environment relationships, and to fit non-linear response trends, respectively. Both the data analysis and graphical output for these analyses were performed using R version 4.4.3 [21].

## 3. Results and Analysis

### 3.1. Insect Species Composition in Different Areas of the PV Power Station

**Figure 3:**
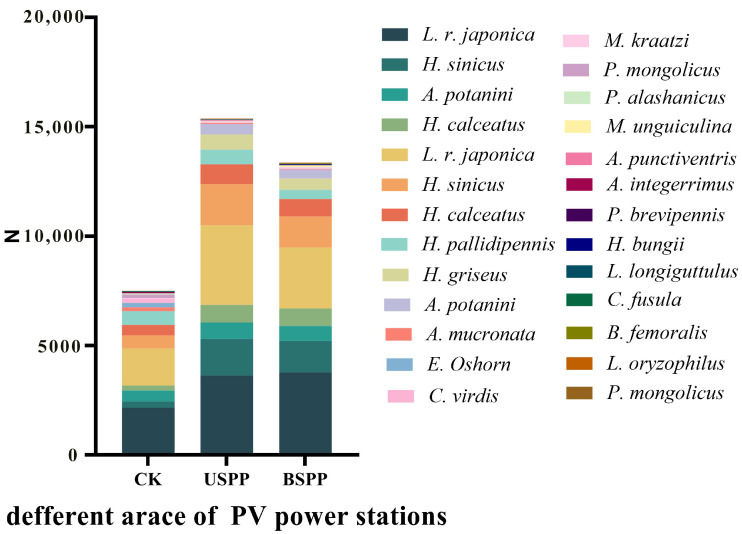
The abundance of insect species in different areas of the PV power station (abundance > 30).

A total of 19,833 insect specimens were collected from different areas of the PV power station, belonging to 23 families and 68 species, including 3 dominant species and 4 common species (Figure 3). The dominant species were the *L. r*. *japonica*, *H*. *sinicus* and *H*. *calceatus*, with 8108, 3905 and 2190 individuals, accounting for 40.88%, 19.69% and 11.04% of the total specimens, respectively. The number of insect species ranked from highest to lowest was: phytophagous insects > predatory insects > saprophagous insects, with 46, 17, and 5 species, making up 67.65%, 25.00% and 7.35% of the total species, respectively. In terms of individual counts, the order was: predatory insects > phytophagous insects > saprophagous insects, with 16,111, 3704 and 18 individuals, accounting for 81.23%, 18.68% and 0.09% of the total specimens, respectively. Outside the PV power station, 4453 insect specimens were collected, with the dominant species being the *L. r*. *japonica*, *H*. *sinicus*, *H. calceatus* and *H. pallidipennis*, totaling 1686, 596, 487 and 628 individuals, representing 37.86%, 13.38%, 10.94% and 14.10% of the specimens in this area. Under the PV panels, 8606 insect specimens were collected, dominated by the *L. r*. *japonica*, *H. sinicus* and *H. calceatus*, with 3638, 1881 and 906 individuals, constituting 42.27%, 21.86% and 10.53% of the specimens in this area. Between the PV panels, 6774 insect specimens were collected, with the *L. r. japonica*, *H. sinicus* and *H. calceatus* being the dominant species, numbering 2784, 1428 and 797 individuals, accounting for 41.10%, 21.08% and 11.77% of the specimens in this area, respectively.

**Figure 4:**
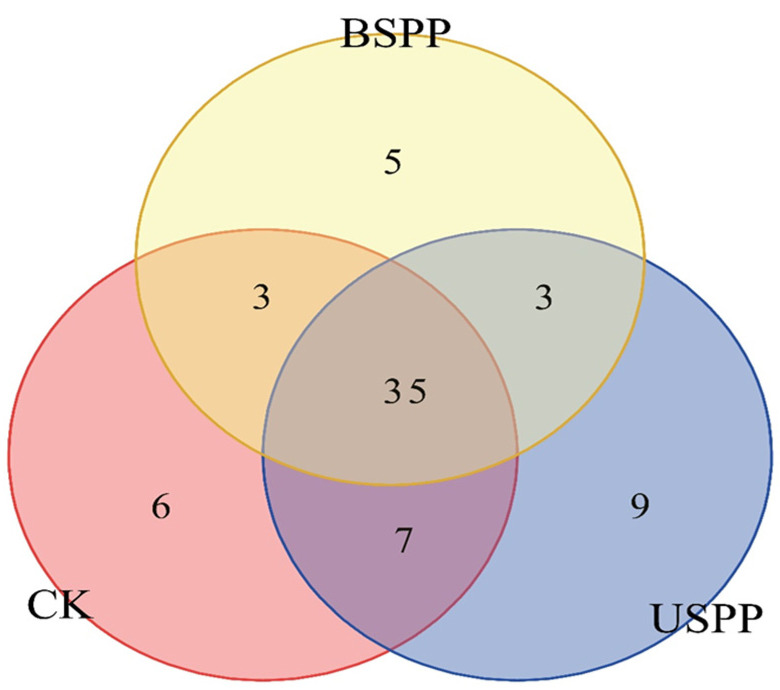
Venn diagram of insect species composition in different areas of the PV power station (at species level).

A total of 51 insect species were collected outside the PV power station, including 6 unique species. Between the PV panels, 46 insect species were collected, with 5 being unique. Under the PV panels, 54 insect species were collected, with 9 being unique. Among the three areas, 35 insect species were shared, encompassing all dominant and common species. The area outside the PV power station and beneath the panels shared 4 species, while the area outside the station and between the panels shared 4 species. Additionally, 7 species were common to both the beneath-panel and between-panel areas (Figure 4).

### 3.2. Insect Diversity in Different Areas of the PV Power Station

#### 3.2.1. Diversity of Insects in Different Areas of the PV Power Station

**Figure 5:**
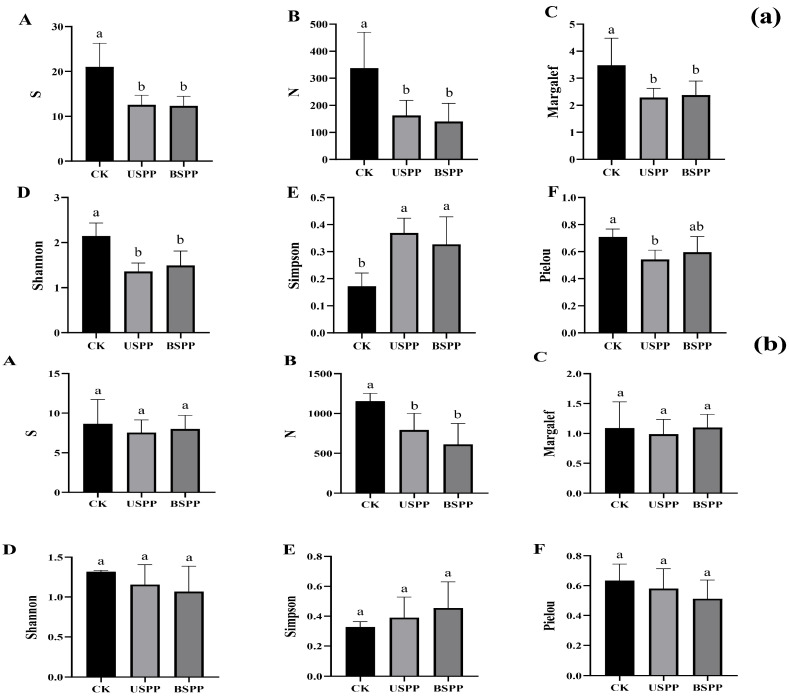
Diversity of insects in different areas of the PV power station. Note: (**a**) phytophagous insect diversity index, (**b**) Predatory insect diversity index, (**A**) is Number of species, (**B**) is Number of individuals, (**C**) is Margalef richness index, (**D**) is Simpson dominance index, (**E**) is Shannon–Wiener diversity index, (**F**) is Pielou evenness index. Different letters on top of the bars indicate significant differences (*p* < 0.05).

The species richness, abundance, Margalef richness index, and Shannon–Wiener index of phytophagous insects were all significantly higher outside the PV power station than inside (both beneath and between PV panels). In contrast, the Simpson dominance index was significantly lower outside the PV power station compared to inside (beneath and between PV panels). Additionally, the Pielou evenness index was significantly higher outside the PV power station than beneath the PV panels (Figure 5a).

The species richness, Margalef richness index, Shannon–Wiener index, Simpson dominance index, and Pielou evenness index of predatory insects did not show significant differences among the different areas of the PV panels. Only the abundance was significantly higher outside the PV power station (CK) than inside (USPP and BSPP) (Figure 5b).

#### 3.2.2. Composition in Different Regions of PV Power Station

**Figure 6:**
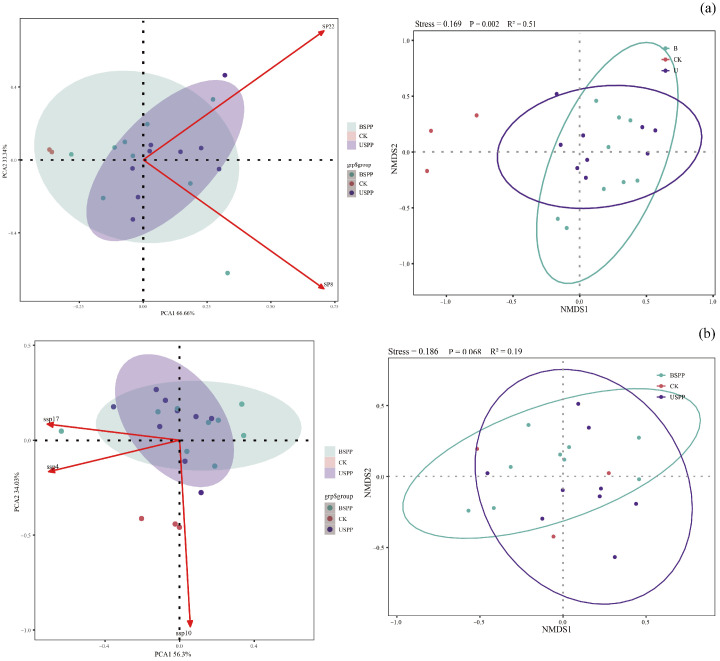
Differences in insect communities across different regions of the PV power station based on PCA and NMDS. Note: (**a**) The red arrows ssp2 and ssp8 represent the dominant species of phytophagous insects, respectively. Differently colored points and ellipses correspond to sample distributions from different regions of the PV power station: USPP (under solar PV panels), BSPP (between solar PV panels), and CK (outside the PV power station). (**b**) Visualization of community differences, where dashed lines represent confidence ellipses for the three PV power station regions, and points represent monitoring sites within each region.

The results of PCA and NMDS showed that the diversity of dominant phytophagous insect species across three different regions exhibited distinct distribution patterns. In the, the clear separation along PC1 (66.66%) and PC2 (33.34%) primarily reflects strong regional niche differentiation. This suggests that the composition of phytophagous insect communities is highly specific to each area within the PV power station. The NMDS analysis further confirmed significant differences between groups (R^2^ = 0.51, *p* = 0.002, Stress = 0.169). This pronounced disparity strongly implies that the assembly of phytophagous insect communities is predominantly governed by deterministic processes, such as the distribution and quality of host plants, which vary significantly across the different habitats created by the PV infrastructure (Figure 6a).

For predatory insects, the PCA showed a lesser degree of separation along PC1 (56.3%) and PC2 (34.02%) compared to phytophagous insects, indicating a more overlapping community structure across regions. Correspondingly, the NMDS analysis revealed a non-significant difference between groups (R^2^ = 0.19, *p* = 0.068, Stress = 0.186), although a trend of dissimilarity is observable. The lack of strong statistical significance suggests that the community composition of predatory insects is more homogeneous across the study area, or that their distribution is influenced by more stochastic factors or smaller-scale microhabitat features rather than broad regional divisions. This contrast with the phytophagous insects aligns with the notion that predators, being more mobile and generalist, respondto a different set of ecological cues than their prey (Figure 6b).

#### 3.2.3. Correlation Analysis Between Predatory and Phytophagous Insect Diversity in Different Areas of the PV Power Station

**Figure 7:**
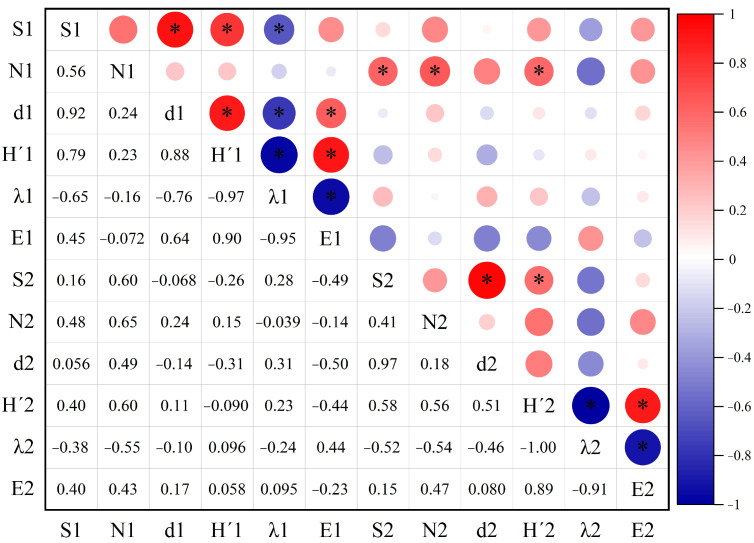
Correlation analysis of diversity between predatory and phytophagous insects in different areas of the PV power station. Note: S1 is species richness of predatory insects, N1 is abundance of predatory insects, d1 is Margalef richness index of predatory insects, H’1 is Shannon–Wiener index of predatory insects, λ1 is Simpson dominance index of predatory insects, and E1 is Pielou evenness index of predatory insects. For phytophagous insects, S2 is species richness of phytophagous insects, N2 is abundance of phytophagous insects, d2 is Margalef richness index of phytophagous insects, H’2 is Shannon–Wiener index of phytophagous insects, λ2 is Simpson dominance index of phytophagous insects, and E2 is Pielou evenness index of phytophagous insects. * is *p* < 0.05.

The species richness, abundance, Shannon–Wiener index, Simpson dominance index, and Pielou evenness index of predatory insects were significantly positively correlated with the abundance of phytophagous insects, while the Simpson dominance index of predatory insects showed a significantly negative correlation with the abundance of phytophagous insects. Additionally, the Shannon–Wiener index of predatory insects was significantly negatively correlated with the Pielou evenness index of phytophagous insects, whereas the Simpson dominance index of predatory insects exhibited a significantly positive correlation with the Pielou evenness index of phytophagous insects (Figure 7).

### 3.3. Response of Insect Diversity to Environmental Factors in PV Power Station

**Figure 8:**
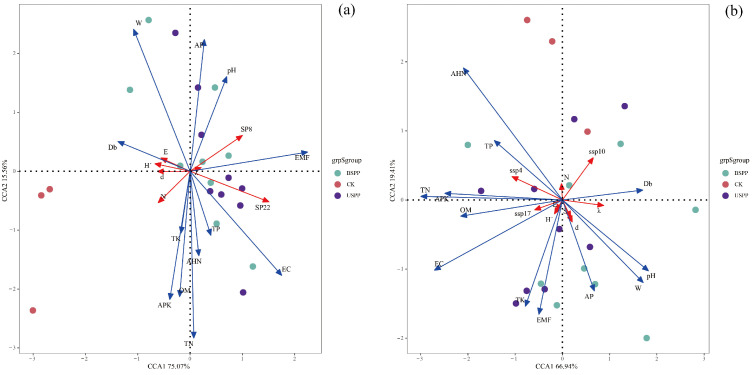
CCA of Insect Communities and Environmental Factors in a PV Power Station. Note: (**a**) Red arrows sp8 (*A. potanini*)and sp22 (*H. griseus*) denote dominant phytophagous insect. Red arrow S is the number of phytophagous insect species, N is the number of phytophagous insect individuals, d is the Margalef richness index of phytophagous insect abundance, H’ is the Shannon–Wiener index for phytophagous insects, λ is the Simpson dominance index for phytophagous insects, and E is the Pielou evenness index for phytophagous insects. Points and ellipses of different colours correspond to sample distributions from distinct areas within the PV power station: USPP (underneath solar PV panels), BSPP (between solar PV panels), and CK (outside the PV power station). Blue arrows: pH is soil pH, OM denotes soil organic, TN denotes total soil nitrogen, TP denotes total soil phosphorus, TK denotes total soil potassium, AHN denotes soil alkali-hydrolysable nitrogen, AP denotes available soil phosphorus, APK is available potassium in soil, EC represents soil leachate electrical conductivity, W indicates soil moisture content, EMF is electromagnetic field strength, and Db measures noise level. (**b**) The red arrows ssp4 (*H. calceatus*), ssp10 (*L. r. japonica*) and ssp17 (*H. sinicus*) represent the dominant species of predatory insects, respectively. Otherwise identical to A. Red arrow S is the number of predatory insect species, N is the number of predatory insect individuals, d is the Margalef richness index of predatory insect abundance, H’ is the Shannon–Wiener index for predatory insects, λ is the Simpson dominance index for predatory insects, and E is the Pielou evenness index for predatory insects. Otherwise identical to (**a**).

The results of the Canonical Correspondence Analysis (CCA) revealed that the combined environmental factors explained 90.63% of the total variation in the phytophagous insect community, with CCA1 and CCA2 accounting for 75.52% and 15.11%, respectively. Soil total nitrogen (TN) and soil water content (W) significantly influenced the diversity indices of the dominant phytophagous species *Anatonochrosis potanini* (Diptera: Canthyloscelididae) (sp8) and *Harpalus griseus* (Coleoptera: Carabidae) (sp22) (*p* < 0.05). Critically, the ordination plot revealed a clear ecological gradient along CCA1, which was strongly correlated with TN and W, indicating that these factors are the primary drivers structuring the community. Specifically, sp8 and sp22 showed a strong positive association with higher soil nitrogen and moisture levels. This pattern strongly supports the dominance of bottom-up control in this system, whereby the distribution and quality of host plants—themselves shaped by TN and W—determine the composition and diversity of the herbivore community (Figure 8a).

The Canonical Correspondence Analysis indicated that environmental factors collectively explained 86.35% of the variation in the predatory insect community, with CCA1 and CCA2 contributing 66.94% and 19.41%, respectively. Soil total nitrogen (TN), hydrolytic nitrogen (AHN), available potassium (APK), and electrical conductivity (EC) significantly influenced the dominant predatory species *H. calceatus* (ssp4), *L. r. japonica* (ssp10), and *H. sinicus* (ssp17) (*p* < 0.05). The ordination results present a more complex picture than that of the phytophagous community, as predators responded to a broader set of factors. The significant roles of APK and EC—indicators of soil heterogeneity and microhabitat conditions—suggest that predator distribution is influenced not only by bottom-up control through prey availability but also by physical habitat structure. This implies that the assembly of predatory insect communities in this managed ecosystem is likely governed by a combination of bottom-up forces and habitat filtering effects (Figure 8b).

### 3.4. Response of Insect Community Diversity in PV Power Station to Environmental Factors

**Figure 9:**
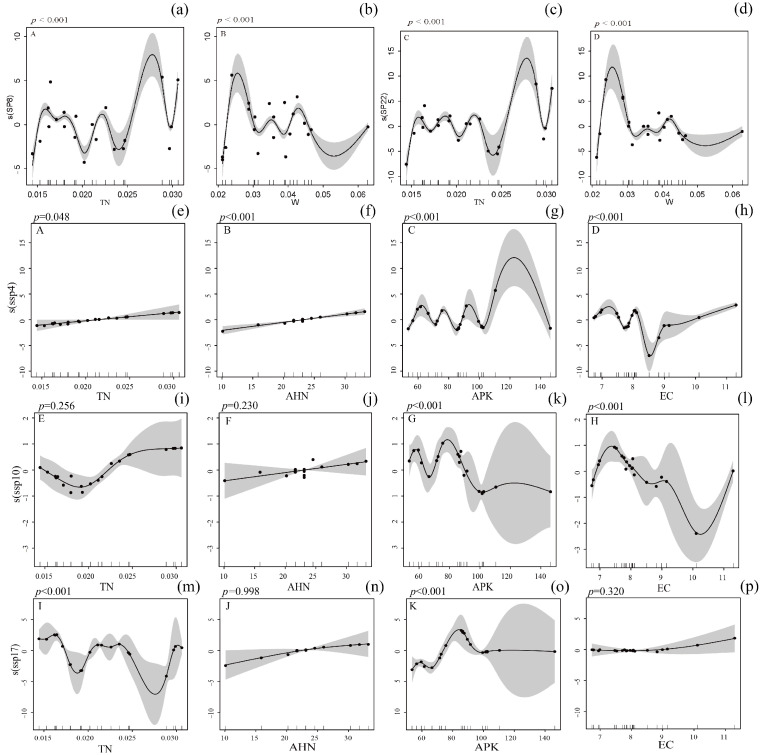
GAM curves of insect diversity in response to environmental factors. Note: (**a**–**d**) represent the relationship between the diversity of phytophagous insect dominant species *A. potanini* (sp8) and *H. griseus* (sp22) with soil total nitrogen (TN) and soil water content (W); (**e**–**p**) represent the relationship between the diversity of predatory insect dominant species *H. calceatus* (ssp4), *L. r. japonica* (ssp10), and *H. sinicus* (ssp17) with soil total nitrogen (TN), alkali-hydrolyzable nitrogen (AHN), soil available potassium (APK), and electrical conductivity (EC).

**Figure A1:**
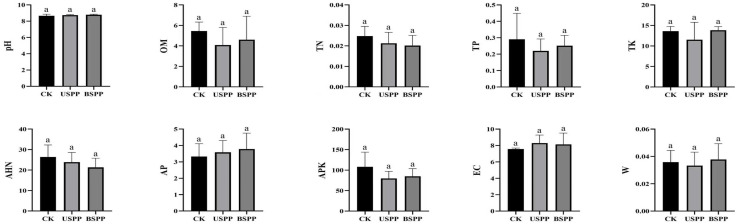
Soil Factors in Different Areas of the Photovoltaic Power Station: pH is soil pH, OM is soil organic matter, TN is total soil nitrogen, TP is total soil phosphorus, TK is total soil potassium, AHN is soil alkali-hydrolysable nitrogen, AP is available soil phosphorus, APK is available potassium in soil, EC is soil leachate electrical conductivity, W is soil water content. The same letters on top of the bars indicate no significant differences.

Among phytophagous insects, GAM revealed that the diversity of the dominant species *A. potanini* (sp8) and *H. griseus* (sp22) showed highly significant nonlinear relationships with soil total nitrogen (TN) and soil water content (W) (*p* < 0.001), while no significant relationships were found with other soil factors including AHN and C/N (*p* > 0.05) (Figure 9a–d and Figure A1). This strongly implies that the distribution of these herbivores is primarily governed by bottom-up forces through host plant quality, with TN and W defining optimal growth conditions for their host plants.

In contrast, among predatory insects, GAM indicated more varied responses. The diversity of *H. calceatus* (ssp4) exhibited a significant linear relationship with TN (*p* < 0.05) and a highly significant linear relationship with AHN (*p* < 0.001), while showing highly significant nonlinear relationships with APK and EC (*p* < 0.001). The relationship with C/N was not significant. Similarly, *L. r. japonica* (ssp10) and *H. sinicus* (ssp17) also showed significant nonlinear relationships with APK and EC (*p* < 0.01), but their diversities were not significantly influenced by TN, AHN, or C/N (Figure 9e–p and Figure A1). This clear divergence from the phytophagous group suggests that predators are responding to a broader set of habitat cues. The consistent importance of APK and EC for all three predator species may indicate that these soil properties are key indicators of microhabitat complexity and prey availability in this managed ecosystem.

## 4. Discussion

### 4.1. Impact of PV Power Station on Insect Diversity

The abundance of both predatory and phytophagous insects was significantly higher outside the PV power station compared to inside (beneath and between PV panels). This finding is consistent with Grodsky et al.’s research in California’s Mojave Desert, which demonstrated that PV power stations reduce vegetation attractiveness to non-bee pollinators and negatively affect local insect populations after 7–8 years of operation [22]. Furthermore, our results indicate that predatory insects accounted for the highest proportion of total individuals, while phytophagous insects represented 66.15% of total species but only 18.81% of total individuals. Predatory insects exhibited stronger responses to environmental factors than phytophagous insects, corroborating Carrara et al.’s findings that high-abundance species show more pronounced reactions to energy availability changes and exhibit stronger correlations with environmental variables [23].

The significant disparity in species diversity between phytophagous and predatory insects, a key finding of this study, presents a compelling ecological phenomenon worthy of in-depth exploration [24]. This pattern is primarily driven by the combined effects of trophic-level energy constraints, resource heterogeneity, and phylogenetic history [25]. According to Lindeman’s “law of 10%” [26], energy transfer between trophic levels follows a geometric attenuation pattern. As primary consumers, phytophagous insects directly utilize approximately 10% of the plant’s net primary productivity, whereas secondary consumers (predatory insects) can access only about 10% of the energy stored in the preceding trophic level [27]. This pyramid-shaped energy distribution fundamentally limits the carrying capacity of higher trophic levels, thereby restricting the species richness of predatory insects [28].

### 4.2. Impact of Environmental Factors in PV Power Station on Insect Community Diversity

This study found that the diversity and abundance of insect communities inside the PV power station were significantly lower than those in the external control areas. This disparity results from the synergistic effects of multiple environmental factors, including alterations in soil properties, electromagnetic radiation, noise pollution, and simplification of vegetation structure. These factors directly or indirectly modify the habitat conditions within the power station, thereby influencing insect community diversity.

The construction and operation of the PV power station have directly altered both the subsurface soil environment and the aboveground vegetation structure. Large-scale foundation installation and site leveling for solar panels lead to increased soil compaction, reduced porosity, diminished water infiltration capacity, and decreased organic matter content [29]. Such physicochemical degradation directly impacts the habitat, reproduction, and overwintering of ground-dwelling beetles, while also indirectly impairing plant growth by altering soil microbial communities and nutrient cycling. More importantly, the shading effect caused by the PV panels creates heterogeneous distributions of light, water, and nutrients, forming a distinct micro-environmental pattern [30]. Additionally, insufficient light under the panels inhibits the growth of heliophilic plants, resulting in vegetation communities dominated by shade-tolerant species, with significantly reduced biomass and coverage. Although areas between panels receive intermittent sunlight, the overall habitat becomes fragmented. As the substrate and food source for insects, the simplification of vegetation structure and loss of diversity directly lead to a sharp decline in the abundance and species richness of phytophagous insects that depend on specific host plants. As the foundation of the food web, the decline of phytophagous insects further triggers bottom-up effects, reducing the food supply for predatory insects and ultimately causing the simplification and collapse of the entire arthropod food web [31].

Meanwhile, electromagnetic radiation and noise generated during the operation of the PV power station also exert potential and profound impacts on insects. Studies have indicated that electromagnetic fields may interfere with the orientation ability of insects that rely on the geomagnetic field for long-distance navigation (such as certain butterflies and moths), disrupting their foraging, migration, and reproductive behaviors [32]. Many insects (such as crickets, cicadas, and mosquitoes) depend on sound for courtship, communication, and predation. Environmental noise may mask these critical acoustic signals, disrupt their reproductive behaviors, and exacerbate imbalances in predator-prey interactions. For instance, noise could make it more difficult for predators to detect prey, while also potentially reducing the ability of prey to sense approaching predators. Such alterations in interactions may have unpredictable effects on community stability [33].

In summary, the impact of PV power station on insect community diversity is a complex process. The core mechanism begins with the direct modification of physical habitats such as soil and vegetation, which reduces the ecosystem’s carrying capacity and resource diversity, severely affecting the survival of specialist insect groups. Subsequently, operational factors such as electromagnetic radiation and noise further disrupt insect physiology and behavior, accelerating the local extinction of sensitive taxa. Ultimately, these impacts are amplified through energy limitations and trophic cascades within the food web, leading to significant changes in the diversity, abundance, and functional structure of the entire insect community. Therefore, future planning and management of PV power station should integrate ecological conservation into the design philosophy, beyond merely considering energy output. Measures such as restoring native vegetation, optimizing sub-panel habitat management, and minimizing anthropogenic disturbances should be implemented to achieve a balance between green energy production and biodiversity conservation.

### 4.3. Limitations and Future Research Directions

This study focuses on the effects of soil properties, electromagnetic fields, noise and vegetation changes induced by PV power station on insect community composition and diversity. Existing research suggests that PV power station may influence local climate through the “PV Heat Island (PVHI)” effect, though findings remain inconsistent: some studies indicate that PV power station in desert areas can lead to a daytime temperature increase of 3–5 °C compared to the surrounding environment, while others report an average daily temperature rise of approximately 0.1 °C [34]. Still, other studies suggest that PV power station act as a “cold source” at night or exhibit cooling effects in summer and warming effects in winter [35], indicating notable seasonal variations and diurnal dynamics. In contrast, the impact of PV power station on humidity demonstrates greater consensus, with multiple studies confirming that air humidity within the station is generally higher than in the surrounding natural environment [36], with increases ranging from 1.15% to 3.93%. Some studies even suggest that large-scale PV power station construction may affect local precipitation by altering regional water cycles [37]. Therefore, future research should systematically integrate meteorological factors such as temperature and humidity, along with their spatiotemporal variations, to more comprehensively and accurately elucidate the mechanisms through which PV power station collectively influence insect communities and their habitat conditions.

## 5. Conclusions

This study examines the impact of PV power on insect community structure and diversity. The results reveal that PV sites host insect communities dominated by a few highly abundant species, with predatory insects exhibiting greater environmental sensitivity. Key environmental drivers include soil nutrients (e.g., total nitrogen, available potassium), which positively correlate with phytophagous insect abundance, while electromagnetic fields and noise pollution negatively affect insect diversity and evenness. Management strategies should prioritize habitat restoration between PV panels and mitigation of noise/electromagnetic field disturbances to minimize ecological impacts. Future research should incorporate microclimate variables to refine impact assessments and enhance conservation planning.

This work provides a foundation for understanding how renewable energy infrastructure modifies arid-land insect communities, highlighting the need for sustainable PV station designs that balance energy production and biodiversity conservation.

## Data Availability

The data presented in this study are available upon request from the corresponding author.

## Abbreviations

The following abbreviations are used in this manuscript:

PV	photovoltaic
USPP	under solar photovoltaic panels
BSPP	between solar photovoltaic panels
CK	outside the photovoltaic power station (control)
S1	species richness of predatory insects
N1	abundance of predatory insects
d1	Margalef richness index of predatory insects
H’1	Shannon–Wiener diversity index of predatory insects
λ1	Simpson dominance index of predatory insects
E1	Pielou evenness index of predatory insects
S2	Species richness of phytophagous insects
N2	abundance of phytophagous insects
d2	Margalef richness index of phytophagous insects
H’2	Shannon–Wiener diversity index of phytophagous insects
λ2	Simpson dominance index of phytophagous insects
E2	Pielou evenness index of phytophagous insects
S	species richness
N	abundance
d	Margalef richness index
H’	Shannon–Wiener diversity index
λ	Simpson dominance index
E	Pielou evenness index
pH	soil pH
OM	soil organic matter
TN	total nitrogen
TP	total phosphorus
TK	total potassium
AHN	alkaline hydrolyzable nitrogen
AP	available potassium
APK	available phosphorus
EC	electrical conductivity of soil leachate
W	soil water content
EMF	electromagnetic field intensity
Db	noise level
Sp	vegetation species richness
H	vegetation height
C	vegetation coverage
F	vegetation frequency
B	vegetation biomass
D	vegetation density
